# Association between anion gap and all-cause mortality of critically ill surgical patients: a retrospective cohort study

**DOI:** 10.1186/s12893-023-02137-w

**Published:** 2023-08-09

**Authors:** Xu Sun, Jianhong Lu, Wenqian Weng, Qiang Yan

**Affiliations:** 1 Department of General Surgery, Huzhou Central Hospital, Affiliated Huzhou Hospital, Zhejiang University School of Medicine, Huzhou, China; 2 Affiliated Central Hospital, Huzhou University, Huzhou, China; 3 The Fifth School of Clinical Medicine, Zhejiang Chinese Medical University, Huzhou, China; 4 Huzhou Key Laboratory of Intelligent and Digital Precision Surgery, Huzhou Central Hospital, Huzhou, China; 5 Department of Intensive Care Unit, Huzhou Central Hospital, Huzhou, China

**Keywords:** Anion gap, All-cause mortality, Surgical intensive care unit, MIMIC-IV

## Abstract

**Background:**

There are few widely accepted and operationally feasible models for predicting the mortality risk of patients in surgical intensive care unit (SICU). Although serum anion gap (AG) is known to be correlated with severe metabolic acidosis, no investigations have been reported about the association between AG level and the outcome during hospitalization in SICU. This study aimed to explore the predictive power of AG for 90-day all-cause mortality in SICU.

**Methods:**

Data of the eligible patients in SICU from 2008 to 2019 was obtained from the Medical Information Mart for Intensive Care IV version 2.0 (MIMIC-IV v2.0) database. Baseline clinical data of the selected patients was compared in different groups stratified by the outcome during their admission via univariate analysis. Restricted cubic spline (RCS) was drawn to confirm the relationship of AG and the short-term mortality. Kaplan-Meier survival curve was plotted in different AG level groups. Univariate and multivariate Cox analyses were performed, and Cox proportional-hazards models were built to investigate an independent role of AG to predict 90-day all-cause mortality risk in SICU. Receiver operating characteristics (ROC) curves analysis was performed to evaluate the predictive value of AG on the 90-day prognosis of patients.

**Results:**

A total of 6,395 patients were enrolled in this study and the 90-day all-cause mortality rate was 18.17%. Univariate analysis showed that elevated serum AG was associated with higher mortality (*P* < 0.001). RCS analysis indicated a positively linear relationship between serum AG and the risk of 90-day all-cause mortality in SICU (χ^2^ = 4.730, *P* = 0.193). Kaplan-Meier survival analysis demonstrated that low-AG group (with a cutoff value of 14.10 mmol/L) had a significantly higher cumulative survival rate than the counterpart of high-AG group (χ^2^ = 96.370, *P* < 0.001). Cox proportional-hazards models were constructed and confirmed the independent predictive role of AG in 90-day all-cause mortality risk in SICU after adjusting for 23 confounding factors gradually (HR 1.423, 1.246–1.625, *P* < 0.001). In the further subgroup analyses, a significant interaction was confirmed between AG and sepsis as well as surgery on the risk for the 90-day mortality. The ROC curve showed that the optimal cut-off value of AG for predicting 90-day mortality was 14.89 with sensitivity of 60.7% and specificity of 54.8%. The area under curve (AUC) was 0.602. When combined with SOFA score, the AUC of AG for predicting 90-day prognosis was 0.710, with a sensitivity and specificity of 70% and 62.5% respectively.

**Conclusions:**

Elevated AG (≥ 14.10 mmol/L) is an independent risk factor for predicting severe conditions and poor prognosis of critical ill surgical patients.

## Background

Critically ill surgical patients are typically admitted to the intensive care unit (ICU) perioperatively attributable to extensive procedures [1], massive hemorrhage [2], systemic inflammatory response syndrome (SIRS) [3], or severe comorbidities [4]. Many of these patients experience hemodynamic instability leading to serious hypoxia and metabolic acidosis [5], which are known to impact patient survival. Therefore, it is important to assess the severity of acid-base disturbance to help predict prognosis and make appropriate management decisions [6].

Serum anion gap (AG) is a crucial parameter that indicates the state of acid-base physiology [7]. High serum AG is often associated with severe acid-base imbalance and poor prognosis in critically ill patients with metabolic acidosis caused by conditions such as sepsis [8], cardiac arrest [9], and kidney dysfunction [10]. However, there are few studies assessing the predictive value of AG for the prognosis of patients admitted to the surgical ICU (SICU). We therefore extracted data from the Medical Information Mart for Intensive Care IV Database version 2.0 (MIMIC-IV v2.0) to evaluate the association between AG and the outcome of critically ill surgical patients.

## Materials and methods

### Data source

This is a retrospect study based on the data retrieved from MIMIC-IV v2.0, which includes more than 70,000 critically ill patients admitted to Beth Israel Deaconess Medical Center (Boston, MA) from 2008 to 2019. One author of the study has passed the Collaborative Institutional Training Initiative (CITI) program course (Certificate NO. 42,303,155) to access the database and obtained the approval from the Institutional Review Boards of Beth Israel Deaconess Medical Center and the Massachusetts Institute of Technology (Cambridge, MA).

The patients ≥ 18 years were included, who were admitted to SICU for the first time. The excluded criteria were (1) SICU stay less than 24 h and (2) AG missing.

### Data extraction

The Structure Query Language (SQL) with PostgreSQL version 10.13 was applied to extract the data from MIMIC-IV about clinical characteristics including age, gender, and comorbidities, and laboratory tests results. The comorbidities included hypertension, diabetes, cirrhosis, acute myocardial infarction (AMI), acute kidney injury (AKI), chronic kidney disease (CKD), chronic obstructive pulmonary disease (COPD), brain injury (cerebral hemorrhage, traumatic brain injury, subarachnoid hemorrhage), acute pancreatitis, sepsis and malignant. The laboratory tests results within the first 24 h were extracted since the admission to SICU, including serum AG, bicarbonate, white blood cell (WBC) count, red blood cell (RBC) count, RBC distribution width (RDW), platelet count, serum creatinine, blood urea nitrogen (BUN), blood glucose, serum calcium, serum magnesium and serum phosphorus. Besides, scoring systems applied in ICU were also recorded, such as sequential organ failure assessment (SOFA) score and simplified acute physiology score II (SAPS II). Most SICU patients have primary diseases related to general surgery, neurosurgery, and orthopedics. Therefore, this study categorizes surgical types into three variables: general surgery, neurosurgery, and orthopedic surgery for analysis.

The diagnosis of sepsis in this paper is based on the diagnostic criteria of sepsis-3.0. That is a suspected or confirmed infection with a SOFA score ≥ 2. [11]

### Study groups and clinical outcome

According to the 90-day outcome during their hospitalization, all the patients included were divided into survival group (n = 5,233) and non-survival group (n = 1,162).

According to the RCS analysis results (AG = 14.10 mmol/L), the patients will be divided into high and low AG groups.

The primary endpoint of the study is all-cause mortality within 90 days since the admission to SICU.

### Statistical analysis

Continuous variables were expressed as mean ± standard deviation (SD) if the data was fitting normal distribution, otherwise they were described as median with interquartile range (IQR). Categorical data were presented as percentage. T-test (for continuous variables with normal distribution), Mann-Whitney U Test (for continuous variables with skewed distribution) and Chi-squared test (for categorical variables) were applied to analyze the difference between the two groups.

Restricted cubic splines (RCS) analysis was used to detect the association between AG and the risk of 90 days all-cause mortality.

Kaplan-Meier analysis was conducted to compare the cumulative survival rate between the high and low AG groups with long-rank test.

Univariable and multivariable Cox regression analyses were performed to examine the relevance between an increased serum AG and the risk of 90-day all-cause mortality in SICU. All the covariates with a P-value < 0.1 when comparing survival group with non-survival group in univariate analysis were selected for multivariable analysis further, evaluating AG to predict the outcome of the critical ill surgical patients. Three regressional models were built and the results were presented as hazard ratios with 95% confidence interval (CI). Model I adjusted for no covariates. In Model II, 12 covariates were adjusted for, including age, SOFA score, bicarbonate, WBC count, RBC count, RDW, platelet count, serum creatinine, BNU, glucose, magnesium and phosphorus. In Model III, eleven more covariates about comorbidity were added based on Model II, including diabetes, cirrhosis, AMI, AKI, CKD, COPD, sepsis, brain injury, malignant tumor, general surgery, and neurosurgery.

Receiver operating characteristics (ROC) curves analysis was performed to evaluate the predictive value of AG on the 90-day prognosis of the patients.

Data analyses were completed via Stata version 14.0 and SPSS version 24.0. Statistical significance was defined as a two-tailed P-value less than 0.05.

## Results

### Subject characteristics

**Fig. 1:**
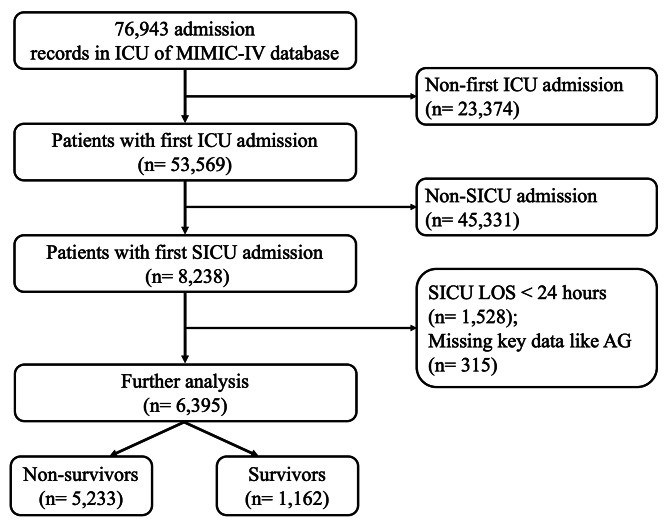
Flowchart of patients screening

6395 eligible patients in SICU were enrolled into this study eventually (Fig. 1), and the clinical characteristics were extracted from MIMIC-IV and summarized in Table 1. In view of the outcome, the patients were divided into survival and non-survival groups. Not surprisingly, the patients in non-survival group were older and with higher comorbidity rate significantly, whose conditions were much more serious with higher SOFA score and SAPSII. In addition, the non-survival group had higher AG, WBC count, RDW, SCR, BUN, glucose, phosphorus and magnesium, but lower bicarbonate, RBC count and platelet count with statistical significance. And there is a difference between two groups in terms of neurosurgery and general surgery (*P* < 0.05). Finally, 23 covariates with a P-value < 0.1 were included for further analysis (Table 1).

**Table 1:** Clinical characteristics of the patients in two groups according to 90-day all-cause mortality

Variables	Total (n = 6,395)		Survival(n = 5,233)	Non-Survival(n = 1,162)		*t/Z/χ* ^*2*^	*P*
Age (year)	63.71 ± 16.75		61.93 ± 16.54	71.75 ± 15.27		-18.552	< 0.001^a^
Female (%)	3080 (48.16)		2516 (48.08)	564 (48.54)		0.080	0.778^b^
SOFA Score	4 (2, 6)		3 (2, 5)	6 (4, 8)		-21.799	< 0.001^c^
SAPS II	33.56 ± 13.44		31.34 ± 12.18	43.54 ± 14.28		-29.893	< 0.001^a^
AG (mmol/L)	14.78 ± 3.82		14.51 ± 3.58	15.59 ± 4.55		-12.036	< 0.001^a^
Bicarbonate (mmol/L)	23.18 ± 4.13		23.33 ± 3.93	22.48 ± 4.85		6.387	< 0.001*
WBC count (×10^9^/L)	10.7 (7.8, 14.3)		10.5 (7.7, 14.0)	11.7 (8.8, 15.8)		-7.716	< 0.001^c^
RBC count (×10^12^/L)	3.78 ± 0.73		3.81 ± 0.71	3.65 ± 0.81		6.615	< 0.001^a^
RDW (%)	14.60 ± 2.07		14.43 ± 1.90	15.35 ± 2.56		-13.950	< 0.001^a^
Platelet count (×10^9^/L)	207 (156, 271)		208 (159, 270)	202 (241, 272)		3.181	0.002^c^
SCR (umol/L)	79.56 (61.88, 106.08)		70.72 (61.88, 97.24)	88.4 (70.72, 141.44)		-11.518	< 0.001^c^
BUN (mg/dL)	16 (12, 25)		15 (11, 22)	22 (15, 35)		-17.084	< 0.001^c^
Glucose (mmol/L)	7.28 (6, 9.06)		7.22 (6, 8.94)	7.58 (6.22, 9.61)		-4.863	< 0.001^c^
Calcium (mmol/L)	2.11 ± 0.21		2.10 ± 0.21	2.11 ± 0.22		-1.131	0.258^a^
Phosphorus(mmol/L)	1.15 ± 0.39		1.14 ± 0.37	1.20 ± 0.48		-4.364	< 0.001^a^
Magnesium (mmol/L)	0.78 ± 0.15		0.78 ± 0.14	0.80 ± 0.16		-4.917	< 0.001^a^
Comorbidity, n (%)							
Hypertension	2971 (46.46)		2407 (46.00)	564 (48.54)		2.467	0.116 ^b^
Diabetes	1548 (24.21)		1241 (23.71)	307 (26.42)		3.792	0.051^b^
Cirrhosis	538 (8.41)		391 (7.47)	147 (12.65)		33.098	< 0.001^b^
CKD	826 (12.92)		613 (11.71)	213 (18.33)		37.006	< 0.001^b^
COPD	586 (9.16)		436 (8.33)	150 (12.91)		23.931	< 0.001^b^
Brain injury	1617 (18.25)		1192 (22.78)	425 (36.57)		95.801	< 0.001^b^
Acute pancreatitis	94 (1.47)		78 (1.49)	16 (1.38)		0.085	0.771^b^
Malignant tumor	1007 (15.75)		773 (14.77)	234 (20.14)		20.637	< 0.001^b^
AMI	118 (1.85)		75 (1.43)	43 (3.70)		26.989	< 0.001^b^
AKI	3400 (53.17)		2570 (49.11)	830 (71.43)		190.195	< 0.001^b^
Sepsis	2932 (45.85)		2240 (42.81)	692 (59.55)		107.415	< 0.001^b^
Surgery, n (%)							
General surgery	709 (11.09)		610 (11.66)	99 (8.25)		9.492	0.002^b^
Neurosurgery	1449 (22.66)		1264 (24.15)	185 (15.92)		36.783	< 0.001^b^
Orthopedic surgery	202 (3.16)		174 (3.33)	28 (2.41)		2.605	0.107^b^
SICU stay (day)	2.58 (1.67, 4.98)		2.38 (1.61, 4.60)	3.43 (1.96, 7.58)		-12.030	< 0.001^c^

Note: a: t-test; b: Chi-squared test (χ²-test); c: Mann-Whitney U Test (W-test). *P* < 0.05 was considered statistically significant

Abbreviation: SOFA: Sequential Organ Failure Assessment; SAPS II: Simplified Acute Physiology Score II; AG: anion gap; WBC: white blood cell; RBC: red blood cell; RDW: red blood cell distribution width; SCR: serum creatine; BUN: blood urea nitrogen; CKD: chronic kidney disease; COPD: chronic obstructive pulmonary disease; AMI: acute myocardial infarction; AKI: acute kidney injury; SICU: surgical intensive care unit

### Serum AG levels and all-cause mortality

Resulting from RCS analysis, a liner relationship was confirmed between serum AG and the risk of 90-day all-cause mortality of the patients in SICU after adjusting for the very 23 covariates (χ^2^ = 4.730, *P* = 0.193).

**Fig. 2:**
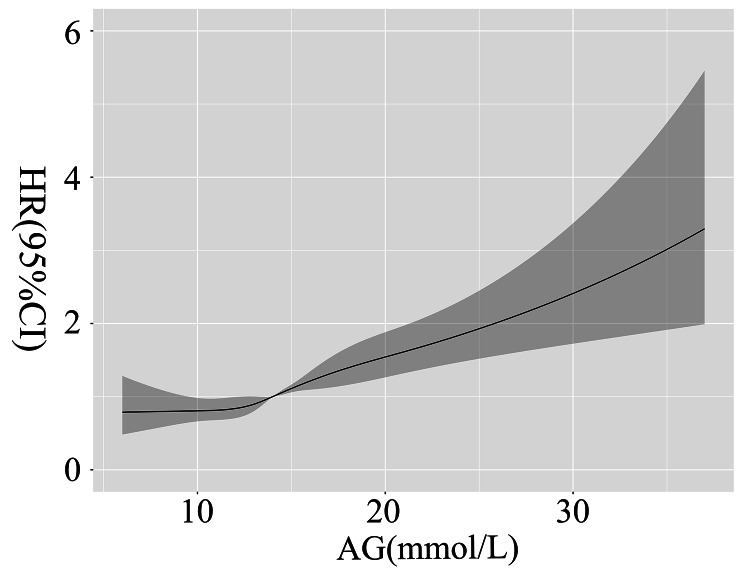
RCS analysis confirmed a liner relationship between serum AG and the risk of 90-day all-cause mortality of the patients in SICU.

All-cause mortality rate was raised with the increased level of AG. As shown in Fig. 2, above the cut-off of 14.10 mmol/L, the higher AG level was, the more risk of the all-cause death the critical ill surgical patients suffered.

### Kaplan-Meier survival curve analysis

**Fig. 3:**
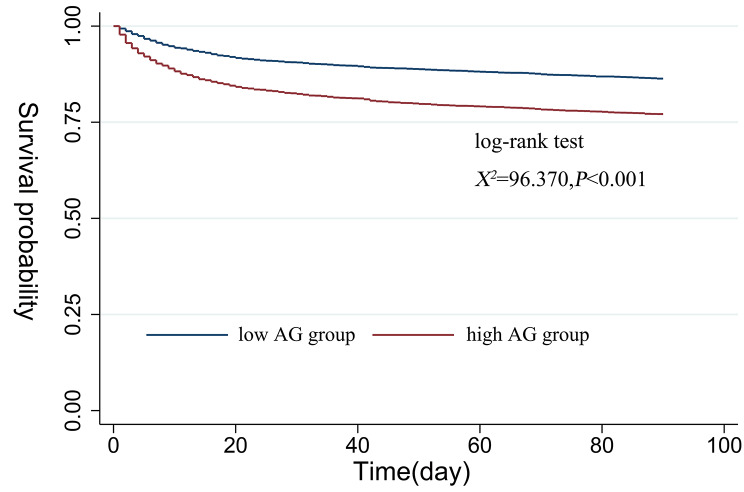
Kaplan-Meier survival curve showed a lower cumulative survival rate for high AG group during hospitalization in SICU.

Following the RCS analysis, the patients were divided into two groups, high-AG (≥ 14.10 mmol/L, n = 3,323) and low-AG (< 14.10 mmol/L, n = 3,072). Indeed, it was presented that low-AG group had a significant higher cumulative survival rate than the counterpart of high-AG group after the survival analysis (χ^2^ = 96.370, *P* < 0.001) (Fig. 3).

### Correlation between AG and the risk of all-cause mortality during hospitalization in critical ill surgical patients

As AG might be a potential to predict the prognosis of the patients in SICU, Cox regression analyses were performed between AG and the risk of 90 days all-cause mortality in SICU. Univariate Cox regression analysis presented that high AG (≥ 14.10 mmol/L) was associated with all-cause mortality within 90 days (HR 1.789, 95% CI 1.590–2.014, *P* < 0.001) (Table 2. Model 1). Based on Model 1, Model 2 adjusted for 12 variables, including age, SOFA score, WBC count, RBC count, RDW, platelet count, bicarbonate, SCR, BUN, glucose, phosphorus and magnesium. And it still maintained the finding that high AG contributed to the 90-day mortality (HR 1.476, 95% CI 1.294–1.684, *P* < 0.001). For the various comorbidities and surgical types of the patients admitted to SICU, whose heterogeneity maybe caused the selection bias, 9 more comorbidity and 2 surgery variables involved were adjusted for on the basis of Model 3. A stable outcome was presented repeatedly, high AG (≥ 14.10 mmol/L) group had a higher 90-day all-cause mortality (HR 1.490, 1.246–1.625, *P* < 0.001) (Table 2. Model 3).

**Table 2:** Cox proportional regression analysis for the association between AG level and 90-day all-cause mortality in SICU.

	Model 1				Model 2					Model 3	
	HR	95%CI	*P*		HR	95%CI	*P*		HR	95%CI	*P*
Low-AG group (n = 3323)	1				1				1		
High-AG group (n = 3072)	1.789	1.590–2.014	< 0.001		1.476	1.294–1.684	< 0.001		1.423	1.246–1.625	< 0.001

Model 1 adjusted for: none

Model 2 adjusted for: age, SOFA score, WBC count, RBC count, RDW, platelet count, bicarbonate, SCR, BUN, glucose, phosphorus and magnesium

Model 3 adjusted for: diabetes, cirrhosis, CKD, COPD, malignant tumor, AMI, AKI, sepsis, brain injury, general surgery, and neurosurgery

Abbreviation: HR: hazard ratio; CI: confidence interval

### Subgroup analyses

**Fig. 4:**
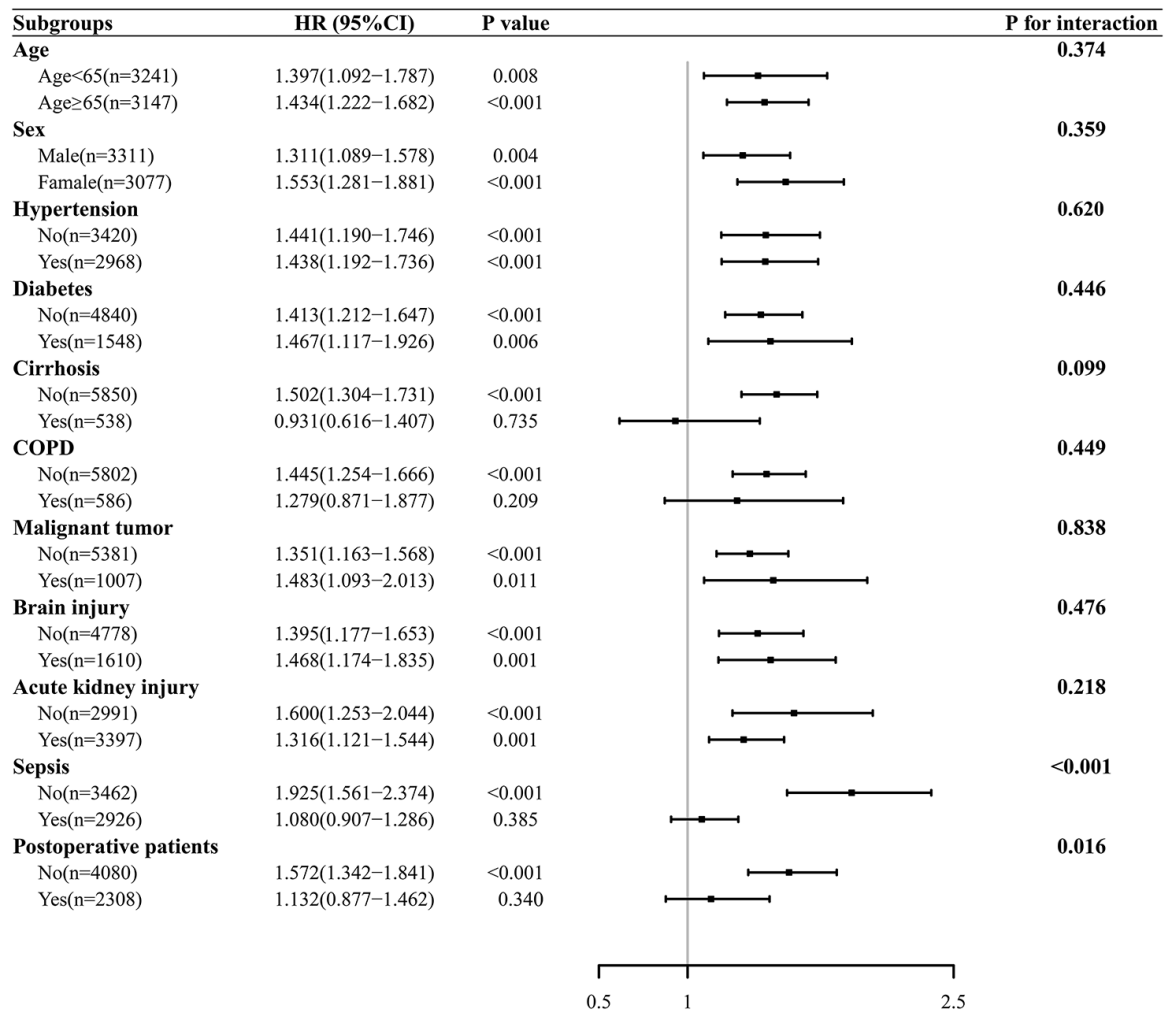
Interactions between AG and the other variables were investigated during the subgroup analyses

Subgroup analyses were performed to investigate the interaction between AG and the variables which were supposed to play a role in the mortality of SICU patients. A significant interaction was confirmed between AG and sepsis on the risk for the 90-day mortality of SICU patients. Non-septic patients would experience a higher risk of mortality following an increased AG level. Besides, there was a significant interaction between AG and surgery on the risk of 90-day mortality (Fig. 4).

### Analysis of ROC curves

**Fig. 5:**
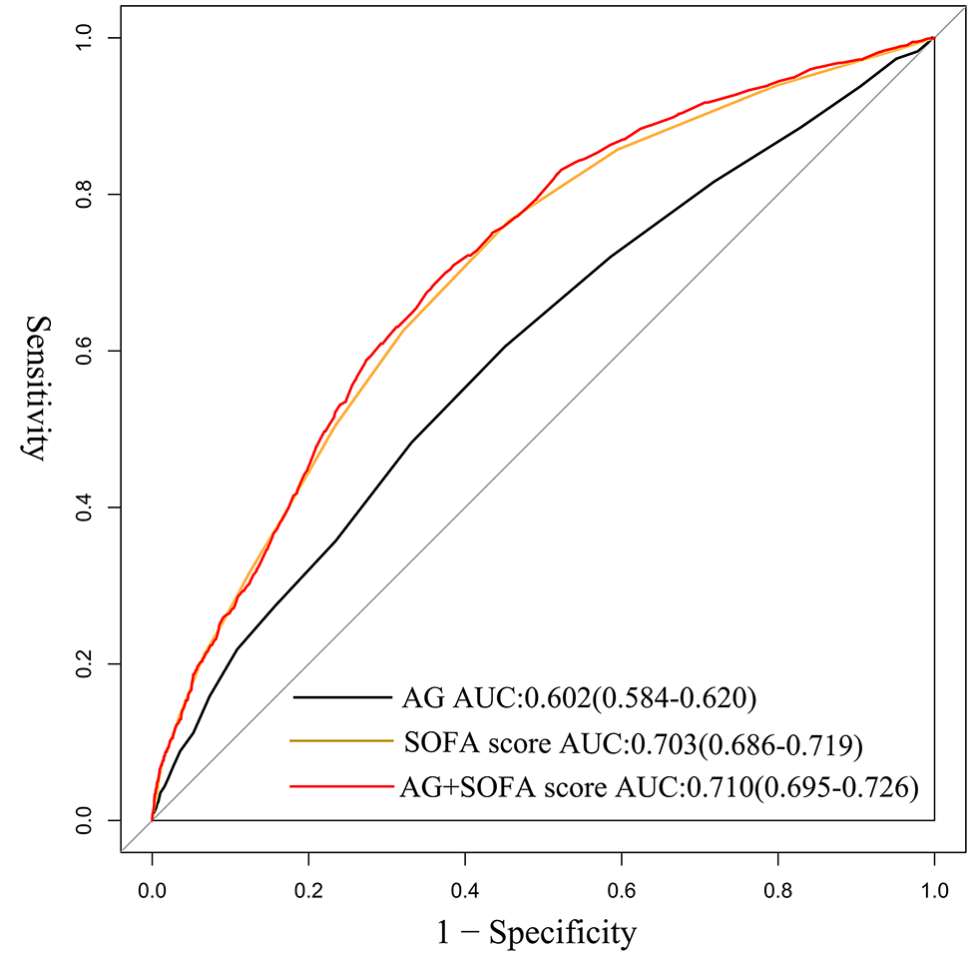
ROC curves for predicting the 90-day mortality in SICU patients

The ROC curve showed that the optimal cut-off value of AG for predicting 90-day mortality was 14.89 with sensitivity of 60.7% and specificity of 54.8%. The area under curve (AUC) was 0.602. When combined with SOFA score, the AUC of AG for predicting 90-day prognosis was 0.710, with a sensitivity and specificity of 70% and 62.5% respectively (Tables 3 and 4; Fig. 5).

**Table 3:** Comparison of the prognostic performance of AG, SOFA score and the combined indicator in patients

Variables	AUC	Cut-off	Sensitivity	Specificity	PPV	NPV	PLR	NLR
AG	0.602	14.89	0.607	0.548	0.230	0.863	1.342	0.718
SOFA score	0.703	3.500	0.766	0.545	0.272	0.913	1.682	0.430
AG + + SOFA score	0.710	0.156	0.700	0.625	0.293	0.904	1.867	0.480

AG, anion gap; SOFA, sequential organ failure assessment; AUC, area under the curve; PPV, positive predictive value; NPV, negative predictive value; PLR, positive likelihood ratio; NLR, negative likelihood ratio

**Table 4:** Comparison of AUCs for predicting 90-day all-cause mortality

Variables	AUC (95% CI)	*P1*	*P2*
AG	0.602 (0.584–0.620)		< 0.001
SOFA score	0.703 (0.686–0.719)	< 0.001	
AG + SOFA score	0.710 (0.695–0.726)	< 0.001	< 0.001

AUC, area under the curve; CI, confidence interval; AG, anion gap; SOFA, sequential organ failure assessment; *P1*, P-value for the equality compared to AG; *P2*, P-value for the equality compared SOFA score

## Discussion

Patients in SICU typically require advanced treatment and monitoring resulting from critical illness or extensive surgery [12, 13]. Because of the severity and complexity of their conditions, these patients are often in a life-threatening state, and therefore, have a poor prognosis [14]. Most of them experience multiple organ dysfunction syndrome (MODS), which makes their vital signs unstable and prone to serious complications [15]. While various factors from the underlying conditions impact the survival of SICU patients, there is a lack of widely accepted and operationally feasible indicators for predicting mortality risk in these patients [16, 17].

As a biochemical parameter, AG has been widely used in the assessment of acid-base balance, electrolyte imbalance, and metabolic abnormalities. Researches have indicated that elevated AG level is associated with the severity and mortality rate of various diseases, such as chronic kidney disease [18, 19], sepsis [20], stroke [21, 22], and sudden cardiac arrest [9]. Recently, researchers have also investigated the relationship between AG and surgical diseases. In a large-scale cohort study, Li and colleagues discovered that postoperative AG levels in cardiac surgery patients were positively associated with short-term and long-term mortality rates and represented an independent risk factor for all-cause mortality [23]. Another study demonstrated that serum AG levels were a significant prognostic factor for mortality in ICU patients who underwent open surgery for aortic aneurysm. Within a specific range, an increase in AG levels corresponded to an increased risk of death [24]. A separate study discovered that patients undergoing thoracic surgery with ∆AG ≥ 7 mmol/L were deemed to be at high risk of death (OR = 4.23, 95% CI: 1.22–14.63, *P* = 0.023) and had a certain predictive value for mortality [25]. Nevertheless, research on the correlation between AG and the entire SICU patient population is presently inadequate. This study observed that AG had the capacity to predict the outcome of patients in SICU within 90 days. In Kaplan-Meier survival curve analysis, the patients in high AG group had a significantly lower survival rate than those in the low group. High AG above the cutoff value of 14.10 mmol/L positively increased the risk of 90-day all-cause mortality. The results suggested that AG may be a potential indicator for predicting the prognosis of SICU patients.

AG reflects the severity of metabolic acidosis in critically ill patients, but is not limited to a specific disease, making it suitable for the complex and diverse diseases of SICU patients. However, as a result, the association between AG and all-cause mortality within 90 days in SICU patients is also influenced by individual differences among patients. Given the numerous factors affecting the prognosis of SICU patients, 21 variables were controlled for in the Cox regression analysis. It was found that the adjusted model still had a good ability to predict the risk of the outcome. Also, ROC analysis was also used to evaluate the efficacy of AG in predicting the 90-day prognosis of SICU patients. Finally, conclusion is obtained that AG has been shown to be a reliable predictor of all-cause mortality risk in SICU patients within 90 days, consistent with the description previously. So, nurses and clinicians should identify high-risk patients early and be vigilant.

Compared with previous studies, this study has some innovations and advantages. Firstly, it is the first one to investigate the correlation between serum AG and in-hospital mortality risk in SICU patients. Secondly, the study provided an indicator, AG, which can be easily obtained and utilized in various clinical settings, including economically underdeveloped areas. What’s more, a convincing conclusion has been obtained after a large-scale study of 6395 eligible patients from the MIMIC-IV database, demonstrating the potential of AG to predict SICU patient prognosis within relatively long-term (90 days) and optimize the specific management. At the same time, this study provides a new indicator and threshold for clinicians and nurses to judge the prognosis of patients. However, this was a retrospective observational study from a single center, and all laboratory tests results were collected only once after the patients’ admission to SICU, without monitoring changes over time. As a result, some potential critical factors during treatment may have been overlooked. Additionally, after subgroup analyses carried out, an interaction between AG and sepsis or cirrhosis was revealed, which was required to be demonstrated in-depth. To address these limitations, further large-scale, multi-center prospective studies are necessary.

## Conclusions

Conclusively, higher serum AG is associated with all-cause mortality of patients in SICU. Elevated AG (≥ 14.10 mmol/L) is an independent risk factor for predicting severe conditions and poor prognosis of critical ill surgical patients.

## Data Availability

The data that support the findings of this study are available from the Medical Information Mart for Intensive Care IV Database version 2.0 (MIMIC-IV v2.0) but restrictions apply to the availability of these data, which were used under license for the current study, and so are not publicly available. Data are however available from the authors upon reasonable request and with permission of the Institutional Review Boards of Beth Israel Deaconess Medical Center and the Massachusetts Institute of Technology.

## Abbreviations

SOFA: Sequential Organ Failure Assessment
SAPS II: Simplified Acute Physiology Score II
AG: anion gap
WBC: white blood cell
RBC: red blood cell
RDW: red blood cell distribution width
SCR: serum creatine
BUN: blood urea nitrogen
CKD: chronic kidney disease
COPD: chronic obstructive pulmonary disease
AMI: acute myocardial infarction
AKI: acute kidney injury
SICU: surgical intensive care unit
LOS: length of stay
RCS: restricted cubic splines
HR: hazard ratio
CI: confidence interval
